# Supplementary material for: Rapid Evolution of Primate Type 2 Immune Response Factors Linked to Asthma Susceptibility
Source: Genome Biol Evol. 2017 Jul 6;9(6):1757–65. doi: 10.1093/gbe/evx120 (PMC5569703; doi:10.1093/gbe/evx120)
Supplement: Supplementary Figures [file evx120_SuppFig1.pdf]

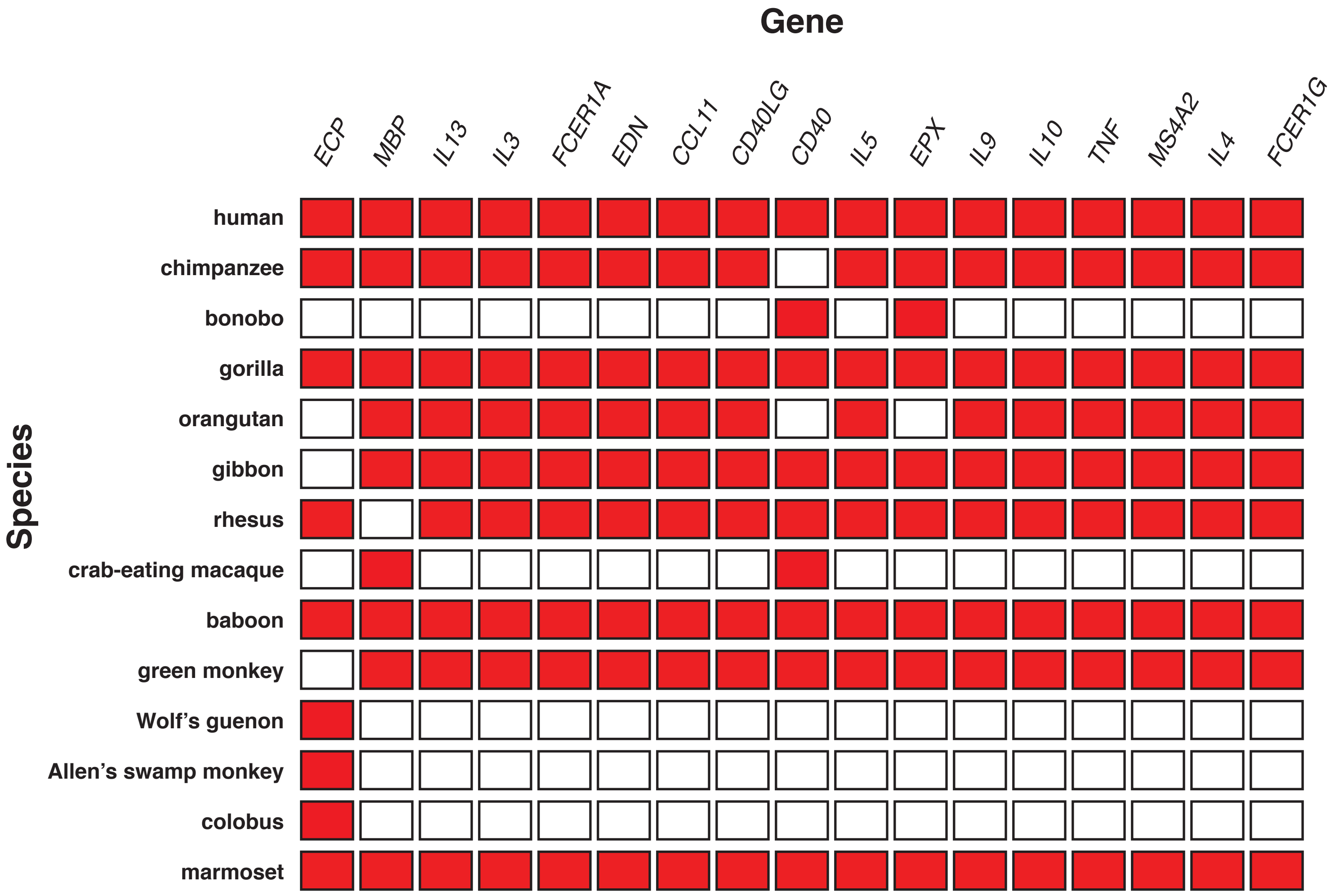

Supplementary Figure 1. Primate species used in initial screen for evidence of positive selection. Red boxes indicate species for which a gene ortholog was included for phylogenetic analysis.

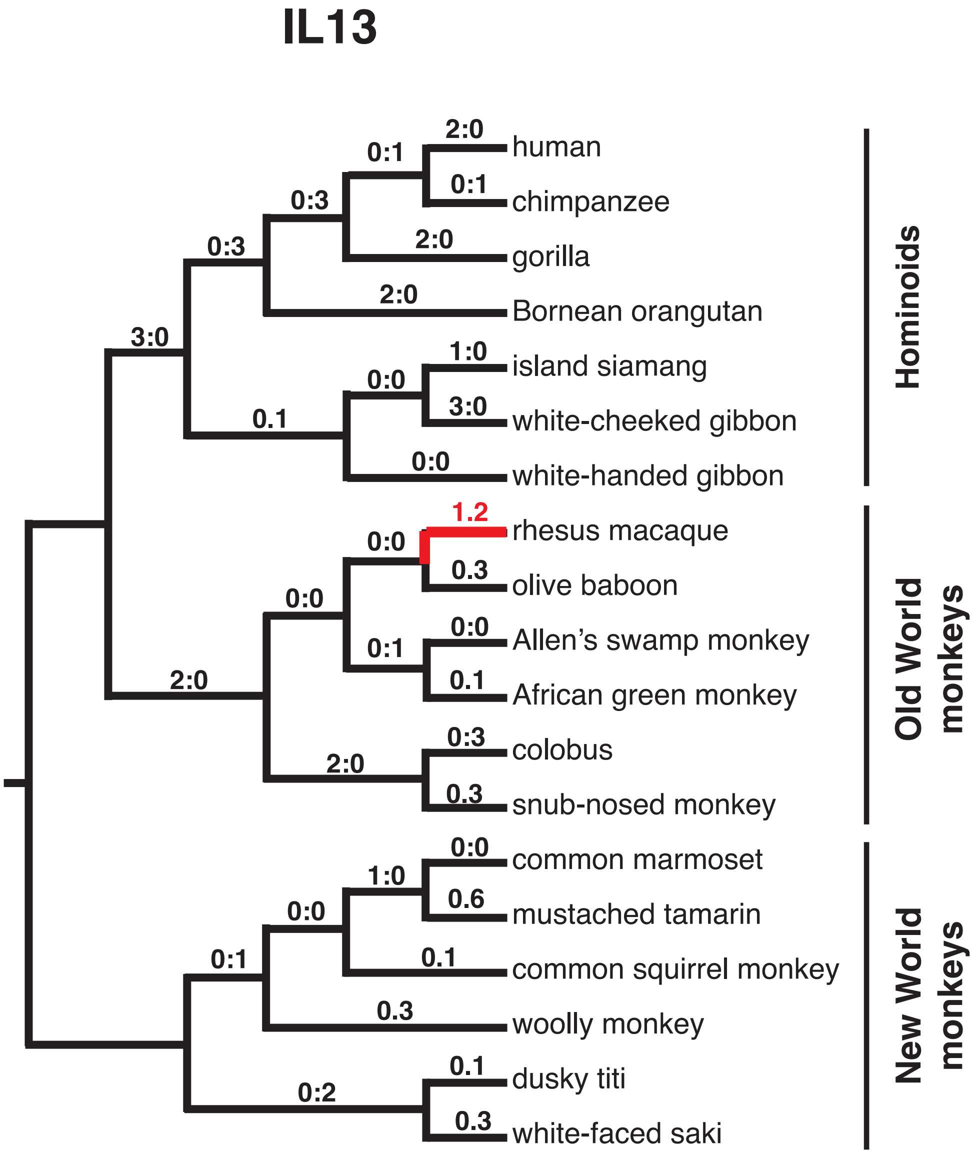

Supplementary Figure 2. dN/dS ratios for IL13 orthologs in the pirmate lineage as calculated using PAML. dN/dS ratios greater than 1 are denoted in red.

ECP

```
human          MVPKLFTSQICLLLLLGLMGVEGSLHARPPQFTRAQWFAIQHISLNPPRCTIAMRAINNY
chimpanzee     MVPKLFTSQICLLLLLGLMGVEGSLHARPPQFTRAQWFAIQHISLNPPRCTIAMRVINNY
bonobo         MVPKLFTSQICLLLLLGLSGVGGSLHAKPRQFTRAQWFAIQHVSLNPPQCTTAMRVINNY
gorilla        MVPKLFTSQICLLLLLGLMGVEGSLHARPPQFTRAQWFAIQHISLNPPRCTIAMRVINNY
orangutan      MVPKLFTSQICLLLLLGLSGVGGSLHAKPRQFTRAQWFAIQHVSLNPPQCATAMRVINNY
               ****************** ** ***** * ************ ***** *  *** ****

human          RWRCKNQNTFLRTTFANVVNVCGNQSIRCPHNRTLNNCHRSRFRVPLLHCDLINPGAQNI
chimpanzee     RWRCKNQNTFLRTTFANVVNVCGNQSIRCPHNRTLNNCHQSRFRVPLLHCDLINPGAQNI
bonobo         QRRCKDQNTFLRTTFANVVNVCGNPNITCPRNRTLHNCHRSRFQVPLLHCNLTNPGAQNI
gorilla        RWRCKNQNTFLRTTFANVVNVCGNQSIRCLHNRTLNNCHRSRFRVPLLHCDLINPGAQNI
orangutan      QRRCKDQNTFLRTTFANVVNVCGNPNITCPSNRSRNNCHHSGVQVPFIYCNLTTPSPQNI
                 *** ******************  * *  **   *** *   **   * *  *  ***

human          SNCTYADRPGRRFYVVACDNRDPRDSPRYPVVPVHLDTTI
chimpanzee     SNCRYADRPGRRFYVVACDNRDPRDSPRYPVVPVHLDATI
bonobo         SNCKYADRTERRFYVVACDNRDPRDSPRYPVVPVHLDTTI
gorilla        SNCRYADRPGRRFYVVACDNRDPQDSPRYPVVPVHLDTTI
orangutan      SNCSYANITGRRFYIVACDNRDPRDSPQYPVVPVHLDTII
               *** **    **** ******** *** *********  *
```

Supplementary Figure 3. Amino acid alignment of great ape ECP homologs. Divergent sites relative to human ECP are highlighted.
