# Supplementary material for: Rapid Evolution of Primate Type 2 Immune Response Factors Linked to Asthma Susceptibility
Source: Genome Biol Evol. 2017 Jul 6;9(6):1757–65. doi: 10.1093/gbe/evx120 (PMC5569703; doi:10.1093/gbe/evx120)
Supplement: Supplementary Tables [file evx120_SuppTables1.pdf]

Table S1. Likelihood ratio test statistics for models of variable selection along branches of the primate IL13, MBP, and ECP phylogenies using PAML.

	lnL	2δ	Df*	p-value
**IL13**				
Model 0<br>Same dN/dS for all branches	-1678.8			
Model 1<br>Different dN/dS ratio for different branches	-1557.4	242.8	35	<0.0001
**MBP**				
Model 0<br>Same dN/dS for all branches	-2211.5			
Model 1<br>Different dN/dS ratio for different branches	-2192.8	37.4	35	0.54
**ECP**				
Model 0<br>Same dN/dS for all branches	-1698.8			
Model 1<br>Different dN/dS ratio for different branches	-1672.9	51.7	26	0.084

* degrees of freedom, equal to one less than the total number of branches in the phylogeny.

Table S2. Summary of positive selection in primate IL13, MBP, and ECP using PAML. Primate species phylogenies were used for all analyses except where indicated for ECP.

	Codon freq.	*M1-M2*		*M7-M8*		Tree length	dN/dS (%)
		**2δ**	**p-value**	**2δ**	**p-value**		
**IL13**	f3x4	0	1	10.1	0.0062	18.77	7.5 (2.2%)
	f61	0	1	<0.0001	>0.99	6.48	1.0 (<0.1%)
**MBP**	f3x4	28.9	<0.0001	29.4	< 0.0001	1.17	3.7 (18.0%)
	f61	32.1	<0.0001	32.2	<0.0001	1.14	4.0 (16.7%)
**ECP**	f3x4	78.7	< 0.0001	81.1	< 0.0001	1.45	5.8 (31%)
	f61	83.1	< 0.0001	83.1	< 0.0001	1.39	6.5 (26.8%)
**ECP (ML gene tree)**	f3x4	34.9	< 0.0001	37.6	< 0.0001	1.07	3.5 (48.1%)
	f61	36.2	< 0.0001	37.5	< 0.0001	1.03	3.6 (47.7%)

Table S3. IL13 log likelihood scores and parameter estimates for four models of variable ω among sites assuming the f3x4 model of codon frequencies using PAML. Amino acid positions shown are for human IL13.

Site model	Parameter estimates	Sites* with ω** > 1	lnL
**M1: neutral**	($\omega 0$=0.04) $f0$= 0.31<br>($\omega 1$=1) $f1$= 0.69<br>branch ω (mean)= 0.71		-1557.4
**M2: selection**	(ω_0=0.04) f_0= 0.31<br>(ω_1=1) f_1= 0.44<br>(ω_2=1) f_2=0.25<br>branch ω (mean)= 0.70	Q130	-1557.4
**M7: β**	p= 0.508<br>q= 0.949<br>branch ω (mean)= 0.35		-1553.5
**M8: β and ω**	p= 0.574<br>q= 1.429 ($f0$= 0.977)<br>**ω_1= 7.5 (f_1= 0.022)**<br>branch ω (mean)= 0.45	Q130	-1548.4

* posterior probabilities >0.95 by Naïve Empirical Bayes (NEB) analysis
** ω = dN/dS

Table S4. MBP log likelihood scores and parameter estimates for four models of variable ω among sites assuming the f3x4 model of codon frequencies using PAML. Amino acid positions shown are for human MBP.

Site model	Parameter estimates	Sites* with ω** > 1	lnL
**M1: neutral**	(ω0=0.05) *f*0= 0.47<br>(ω1=1) *f*1= 0.53<br>branch ω (mean)= 0.55		-2192.8
**M2: selection**	(ω_0=0.36) f_0= 0.81<br>(ω_1=1) f_1= 0<br>**(ω_2=3.6) f_2=0.18**<br>branch ω (mean)= 0.96	E55<br>S114<br>S119<br>V134<br>I141<br>S150<br>A151<br>R171<br>L213<br>R215	-2178.3
**M7: β**	*p*= 0.0175<br>*q*= 0.0108<br>branch ω (mean)= 0.60		-2193.1
**M8: β and ω**	*p*= 57.0<br>*q*= 99.0 (f_0= 0.820)<br>**ω_1= 3.6 (f_1= 0.18)**<br>branch ω (mean)= 0.96	E55<br>S114<br>S119<br>V134<br>S150<br>A151<br>R171<br>L213<br>R215	-2178.4

* posterior probabilities >0.99 by Naïve Empirical Bayes (NEB) analysis.

** ω = dN/dS

Table S5. Log likelihood scores and parameter estimates for ECP using an accepted species phylogeny for four models of variable ω among sites assuming the f3x4 model of codon frequencies using PAML. Amino acid positions shown are for human ECP.

Site model	Parameter estimates	Sites* with ω** > 1	lnL
**M1: neutral**	($\omega 0=0$) $f0= 0.49$<br>($\omega 1=1$) $f1= 0.51$<br>branch ω (mean)= 0.51		-1672.9
**M2: selection**	($\omega_0=0$) $f_0= 0.44$<br>($\omega_1=1$) $f_1= 0.28$<br>**($\omega_2=6.1$) $f_2=0.28$**<br>**branch ω (mean)= 1.97**	I40<br>N59<br>Y60<br>R61<br>W62<br>Q67<br>G83<br>Q85<br>N140	-1633.6
**M7: β**	$p= 0.0069$<br>$q= 0.0050$<br>branch ω (mean)= 0.60		-1674.3
**M8: β and ω**	$p= 0.0050$<br>$q= 0.0117$ ($f_0= 0.69$)<br>**$\omega_1= 5.8$ ($f_1= 0.31$)**<br>**branch ω (mean)= 2.0**	I40<br>N59<br>Y60<br>R61<br>W62<br>Q67<br>G83<br>Q85<br>R132<br>N140	-1633.7

* posterior probabilities >0.99 by Naïve Empirical Bayes (NEB) analysis

** ω = dN/dS

Table S6. Log likelihood scores and parameter estimates for ECP using a PhyML gene phylogeny for four models of variable ω among sites assuming the f3x4 model of codon frequencies using PAML. Amino acid positions shown are for human ECP.

Site model	Parameter estimates	Sites* with ω** > 1	lnL
**M1: neutral**	($\omega 0=0$) $f0$= 0.40<br>($\omega 1=1$) $f1$= 0.60<br>branch ω (mean)= 0.60		-1549.1
**M2: selection**	($\omega_0=0$) f_0= 0.52<br>($\omega_1=1$) f_1= 0<br>**($\omega_2=3.5$) f_2=0.48**<br>**branch ω (mean)= 1.68**	W62<br>G83<br>R102<br>N140	-1531.6
**M7: β**	p= 0.0050<br>q= 0.0050<br>branch ω (mean)= 0.50		-1550.4
**M8: β and ω**	p= 0.0050<br>q= 99.0 (f_0= 0.52)<br>**ω_1= 3.5 (f_1= 0.48)**<br>**branch ω (mean)= 1.68**	V2<br>I40<br>R61<br>W62<br>Q67<br>G83<br>Q85<br>R102<br>F103<br>H109<br>R132<br>N140<br>T158	-1531.6

* posterior probabilities >0.99 by Bayes Empirical Bayes (BEB) analysis
** ω = dN/dS

Table S7. Summary of positive selection in primate transferrin using MEME and FEL algorithms. Amino acid positions shown are for human transferrin.

Model	Sites with evidence of positive selection (p-value)			
	**IL13**	**MBP**	**ECP (species tree)**	**ECP (gene tree)**
**MEME**	P7 (0.022)<br>G13 (0.059)<br>A16 (0.072)<br>T21 (0.062)<br>I23 (0.071)<br>L25 (0.011)<br>G30 (0.015)<br>P39 (0.068)	P33 (0.092)<br>E55 (0.011)<br>C104 (0.063)<br>I141 (0.035)<br>A151 (0.070)<br>R168 (0.025)<br>R171 (0.061)<br>R209 (0.017)<br>H211 (0.045)<br>L213 (0.082)	V2 (0.077)<br>I40 (0.0099)<br>R61 (0.068)<br>W62 (0.051)<br>R63 (0.081)<br>V78 (0.0023)<br>V79 (0.077)<br>G83 (0.026)<br>Q85 (0.034)<br>R102 (0.054)<br>N140 (0.076)	I40 (0.065)<br>N59 (0.027)<br>R63 (0.034)<br>V78 (0.0026)<br>G83 (0.085)<br>Q85 (0.072)<br>Q118 (0.064)
**FEL**	H103 (0.099)<br>L120 (0.092)	S114 (0.086)<br>A151 (0.051)<br>R171 (0.067)<br>R209 (0.054)<br>L213 (0.061)	R61 (0.036)<br>W62 (0.027)<br>G83 (0.053)<br>Q85 (0.022)	W62 (0.061)<br>Q85 (0.053)

Table S8. Summary of positive selection in IL13, MBP and ECP using FUBAR algorithm. Amino acid positions shown are for human transferrin.

Sites with evidence of diversifying selection	Posterior probability β>α	Empirical Bayes Factor
**IL13**		
L120	0.935	18.4
Q130	0.966	36.8
**MBP**		
E55	0.947	20.5
S114	0.950	22.0
I141	0.928	14.9
151	0.984	72.8
R171	0.963	29.9
188	0.913	12.1
192	0.912	12.0
209	0.933	16.1
L213	0.968	34.8
**ECP (species tree)**		
V2	0.94	15.7
I40	0.98	75.2
N59	0.96	28.6
Y60	0.96	31.1
R61	0.99	133.2
W62	0.99	147.8
R63	0.96	26.9
Q67	0.98	57.9
R72	0.92	11.7
V79	0.92	13.1
G83	0.99	101.2
Q85	0.99	130.9
H91	0.92	12.1
H109	0.90	9.4
Q118	0.90	9.0
N122	0.94	16.0
R132	0.97	33.3
N140	0.96	31.5
T158	0.94	16.8
**ECP (gene tree)**		
V2	0.94	15.9
I40	0.93	13.9
R61	0.93	14.8
W62	0.98	56.5
Q67	0.92	11.9
G83	0.97	35.8
Q85	0.96	28.9

H91	0.91	11.1
R102	0.95	21.7
H109	0.90	9.8
R132	0.92	13.1
N140	0.94	16.5
T158	0.94	17.1

Table S9. BUSTED analysis likelihood ratio test statistics for gene-wide episodic diversifying selection on IL13, MBP and ECP.

IL13

Model	log L	AICc	Tree length	LRT p-value
**Unconstrained**	-1521.28	3152.97	202.4	0.116
**Constrained**	-1523.44	3155.19	267.94	

MBP

Model	log L	AICc	Tree length	LRT p-value
**Unconstrained**	-2168.20	4445.87	1.22	<0.0001
**Constrained**	-2183.50	4474.41	1.09	

ECP

Model	log L	AICc	Tree length	LRT p-value
**Unconstrained**	-1529.50	3148.81	1.10	0.006
**Constrained**	-1534.56	3156.85	0.98	

Table S10. Likelihood ratio test statistics for models of variable selection along branches of the primate IL4R gene phylogeny using PAML with sequences obtained from Genbank (human, chimpanzee, bonobo, gorilla, orangutan, rhesus macaque, crab-eating macaque, pig-tailed macaque, drill, sooty mangabey, African green monkey, golden snub-nosed monkey, colobus, marmoset, squirrel monkey, and night monkey).

	**Codon freq.**	***M1-M2***		***M7-M8***		**Tree length**	**dN/dS (%)**
		**2δ**	**p-value**	**2δ**	**p-value**		
**IL4R**	f3x4	1	0.60	2.5	0.28	0.88	1.2 (<0.28%)

Table S11. Primers used in this study.

Primer	Sequence
IL13-for	GTTGGCACTGGGCCTCATG
IL13-rev	GGTCCTGTCTCTGCAAATAATGATGC
MBP-for	GGAAGGTCTCTGGGTGGGATAAAGCC
MBP-rev	GCCCAGGAGAGGGCAGCTCTGAAC
ECP-for	CAACCAGCTGGATCAGTTCTCACAGG
ECP-rev	GAGGAGCTTGGCAGATGAGTGATGATG
